# Seronegative End-Stage Type I Achalasia With Megaesophagus in a Middle-Aged Woman From Central America: A Case Report

**DOI:** 10.7759/cureus.115407

**Published:** 2026-08-29

**Authors:** Daniel C Dominguez, Jeams Richard, Amr El-Talla

**Affiliations:** 1 Diagnostic Radiology, HCA Healthcare, Miami, USA; 2 Medical School, Nova Southeastern University, Miami, USA

**Keywords:** achalasia, chagas disease, esophageal manometry, esophagram, megaesophagus

## Abstract

Achalasia is a primary esophageal motility disorder defined by impaired lower esophageal sphincter relaxation and absent peristalsis. Late-stage disease can lead to megaesophagus, a severe dilation and tortuosity of the esophageal body. While most cases are idiopathic, secondary achalasia can be caused by Chagas disease (*Trypanosoma cruzi* infection), an important consideration in patients from endemic regions of Central and South America.

Our case highlights a 51-year-old female from Nicaragua who presented to the emergency department with an acute onset of shortness of breath. A computed tomography angiogram (CTA) ruled out a pulmonary embolism but revealed a severely distended esophagus impacted with food contents. A chest radiograph demonstrated cardiac silhouette enlargement and left-base atelectasis. Gastroenterology was consulted, and the patient was admitted. A barium esophagogram revealed a markedly dilated, tortuous esophagus with an abrupt transition point with narrowing of the distal esophagus at the gastroesophageal junction (GEJ), resulting in the characteristic “bird’s beak” appearance. High-resolution esophageal manometry confirmed an elevated median integrated relaxation pressure (IRP) and absent contractility, establishing a diagnosis of achalasia. Because the patient emigrated from a Chagas-endemic region, *T. cruzi* IgG antibody testing was performed twice and returned negative. The patient underwent a successful upper endoscopy with pneumatic balloon dilation of the GEJ. Her respiratory and dysphagia symptoms resolved; she tolerated a regular diet, and she was safely discharged.

This case highlights a unique presentation of end-stage achalasia with megaesophagus initially manifesting as acute dyspnea. It underscores the diagnostic imperative to screen for secondary infectious etiologies like Chagas disease in endemic populations, while recognizing the limitations of single-assay serology. Ultimately, idiopathic achalasia remains the most probable diagnosis once pseudoachalasia and mechanical obstructions are excluded via high-resolution esophageal manometry and endoscopy.

## Introduction

Achalasia is characterized by abnormal peristalsis and impaired relaxation of the lower esophageal sphincter secondary to loss of the normal inhibitory intramural ganglion cells in the myenteric plexus [1]. It is an uncommon esophageal motility disorder with an incidence of roughly 1-2 per 100,000 people/year and typically presents in middle adulthood (30-60 years). It has an equal prevalence between males and females [2]. Common symptoms of achalasia include dysphagia, regurgitation, foul-smelling breath (termed halitosis), and occasional chest pain. The standard clinical presentation of achalasia features progressive dysphagia to both solids and liquids, weight loss, and nocturnal cough or aspiration. Left untreated, chronic retention of ingested material and progressive hydrostatic pressure can result in megaesophagus, described as severe dilatation of the esophagus, as a late-stage complication of achalasia. Megaesophagus can then present with respiratory symptoms via several mechanisms, including aspiration of ingested material trapped in the dilated esophagus, direct compression of the tracheobronchial tree, or compression of adjacent lungs resulting in compressive atelectasis [3].

Diagnostic approaches rely on a multimodality approach with barium esophogram demonstrating the classic “bird’s beak” narrowing of the distal esophagus, endoscopy ruling out structural mimics, and high-resolution manometry confirming the diagnosis. High-resolution manometry can further classify achalasia into three distinct categories based on the Chicago Classification [4]. Type 1 achalasia is defined as complete absence of peristalsis with absence of panesophageal pressurization on esophageal manometry, considered classic achalasia. Type 2 achalasia is defined as complete absence of peristalsis with mild uniform panesophageal pressurization. Type 3 achalasia is defined as uncoordinated, spastic contractions of the lower esophagus with or without panesophageal pressurization. While the majority of achalasia cases are considered primary and idiopathic in etiology, secondary achalasia can arise from malignant infiltration (termed pseudoachalasia) or other infectious/inflammatory causes. Chagas disease, caused by infection with the protozoan parasite *Trypanosoma cruzi*, is a known, well-recognized cause of secondary achalasia [5], especially in South America where the parasite is endemic.

Treatment options aim to reduce lower esophageal sphincter pressure in order to facilitate bolus transit. Temporary treatment options include endoscopic interventions such as botulinum toxin injection or pneumatic dilatation. More definitive treatment options include laparoscopic Heller myotomy or peroral endoscopic myotomy [6]. In patients with end-stage megaesophagus, surgical resection with esophagectomy may be required. This case reports a 51-year-old female from Nicaragua presenting with end-stage Type 1 achalasia with megaesophagus. This case is distinct as the patient’s primary, acute complaint was dyspnea rather than classic gastrointestinal symptoms. Given the patient’s Central American origin, achalasia secondary to Chagas disease was suspected; however, workup revealed a seronegative status. This report highlights the diagnostic pitfalls of atypical, cardiopulmonary presentation of esophageal disease and emphasizes the clinical challenges of managing end-stage idiopathic achalasia.

## Case presentation

Methods

The patient was interviewed, and the electronic medical record was reviewed, including clinical notes, laboratory results, imaging studies, and operative reports. All data were de-identified to ensure patient confidentiality before the case was developed.

Presentation

A 51-year-old female from Nicaragua presented to the emergency department, brought in by Emergency Medical Services (EMS) as a transfer from a nearby hospital for evaluation of acute shortness of breath that started the prior night. The patient denied any history of nausea, vomiting, fever, chest pain, abdominal pain, or weight loss. All vitals were within normal limits. Reported physical exam revealed signs of increased work of breathing with accessory muscle use and nasal flaring. The patient was also found to have dry mucous membranes. Physical exam, including pulmonary auscultation, was otherwise unremarkable. Initial external computed tomography angiogram (CTA) was negative for pulmonary embolism but was reported to show a distended esophagus with impacted ingested contents. Labs, including complete blood count (CBC), comprehensive metabolic panel (CMP), and high-sensitivity troponin, were unremarkable. Initial chest radiograph demonstrated cardiac silhouette enlargement and left base atelectasis with pulmonary congestion (Figure 1). The patient was ultimately admitted for further evaluation.

**Figure 1:**
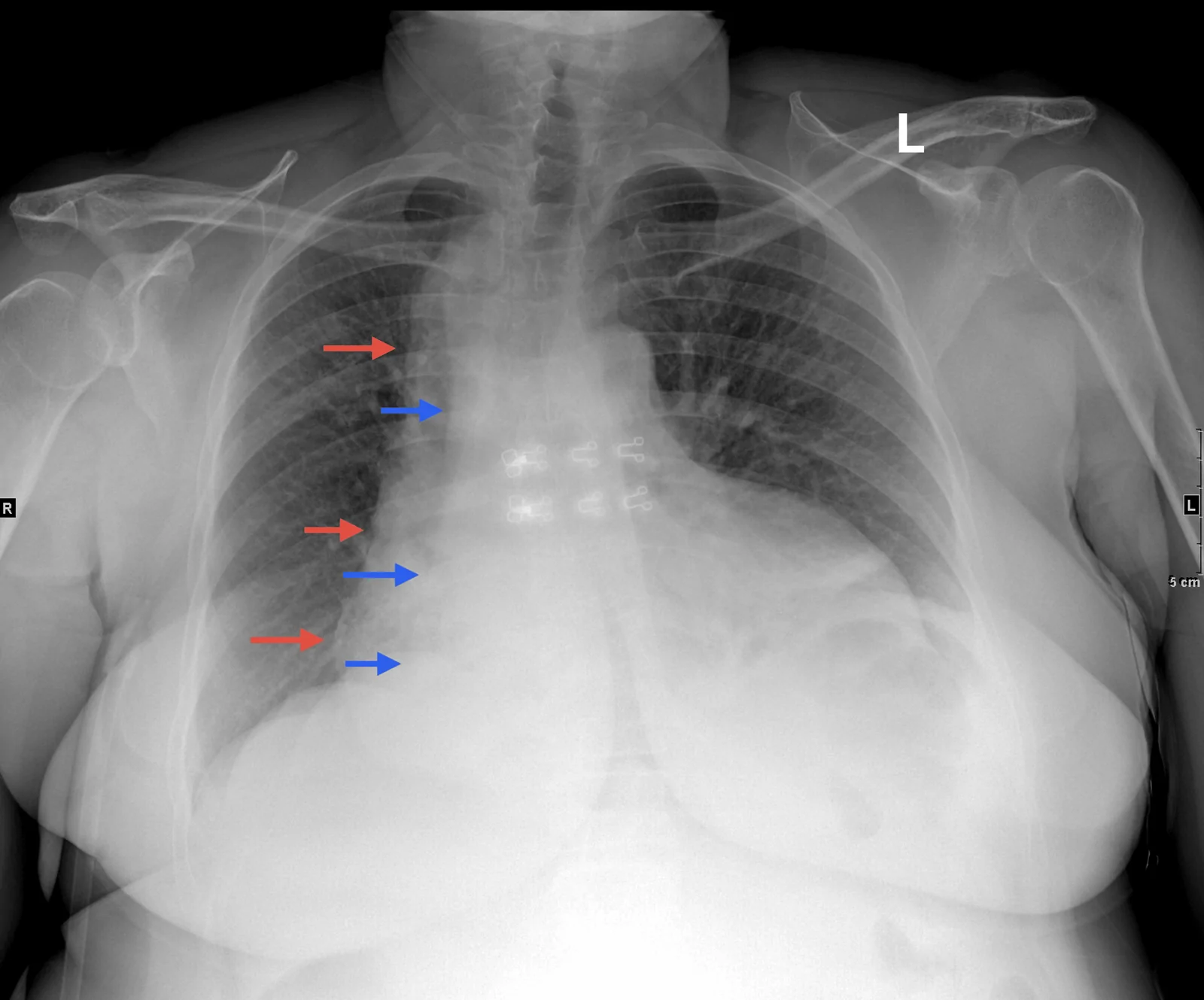
Chest Radiograph A chest radiograph performed in the emergency department demonstrated left basilar streaky consolidation with mild elevation of the left hemidiaphragm, favored to represent atelectasis. Mediastinal widening was also demonstrated, resulting in a “double right heart border” sign, with the true right heart border indicated by the blue arrows and the megaesophagus indicated by the red arrows.

The patient was initially placed on a nil per os (NPO) diet; gastroenterology was consulted, and *T. cruzi* immunoglobulin G (IgG) antibodies were tested using an enzyme-linked immunosorbent assay (ELISA) and found to be negative. *T. cruzi *IgG antibodies utilizing lateral flow assay methodology were also performed and found to be negative (Table 1). Endoscopy was performed on day 2 of patient hospitalization, which demonstrated a severely dilated and tortuous esophagus and significant amounts of ingested material filling the distal esophageal lumen. Subsequent barium esophogram on day 3 also demonstrated a markedly dilated, tortuous esophagus with an abrupt transition point at the level of the gastroesophageal junction (GEJ), giving the classic “bird’s beak” appearance (Figure 2). On day 4, high-resolution esophageal manometry was performed, demonstrating absent contractility in 100% of swallows, with no evidence of panesophageal pressurization, consistent with type I achalasia. The patient underwent endoscopy with pneumatic balloon dilatation on day 6, with significant improvement of symptoms and ability to tolerate a regular diet. A post-treatment barium esophogram was ordered, which the patient ultimately refused, and the patient was discharged home for continued follow-up and definitive treatment on an outpatient basis. A complete timeline of the patient's hospitalization is provided in Table 2.

**Table 1:** Serological Screening for Secondary Infectious Etiology Current recommendations for the diagnosis of Chagas disease generally require two distinct serologic assays using different antigens or methodologies, as no single assay has adequate sensitivity and specificity across all populations [5]. In this patient, a repeated negative *T. cruzi* IgG antibody test using ELISA and LFA methodologies makes Chagas disease highly unlikely, supporting a diagnosis of idiopathic Type 1 achalasia.

Timeline	Laboratory Assay	Methodology	Patient Value	Reference Range	Clinical Interpretation
Day 1	*Trypanosoma cruzi* antibody, IgG	Semi-quantitative Enzyme-Linked Immunosorbent Assay (ELISA)	0.2 IV	≤1.0 IV	Negative (no significant antibody level detected)
Day 2	*T. cruzi* antibody, IgG	Lateral Flow Assay (LFA)	Non-reactive	Non-reactive	Negative (no *T. cruzi* antibodies detected)

**Figure 2:**
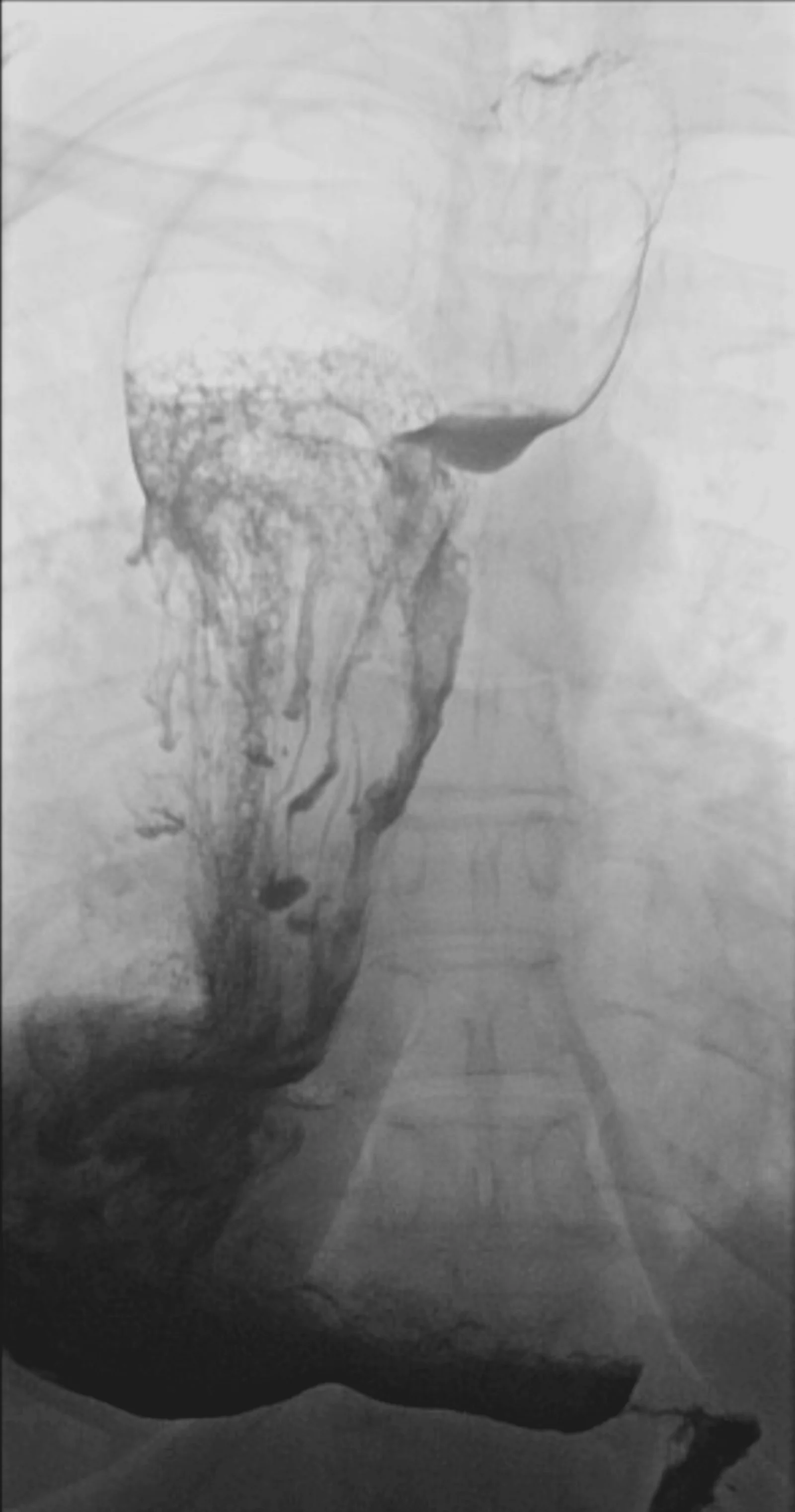
Barium Esophagram Barium esophogram demonstrates a severely dilated and tortuous esophagus, consistent with megaesophagus. Abrupt narrowing of the distal esophagus at the level of the gastroesophageal junction is visualized in the right lower quadrant of this image, resulting in the classic “bird’s beak” appearance.

**Table 2:** Timeline of Clinical Events and Interventions NPO: Latin for “nothing by mouth”, no oral intake diet; GI: gastroenterology; EGD: endoscopy; GEJ: gastroesophageal junction; OD: outer diameter; HrEM: high-resolution esophageal manometry; IRP: integrated relaxation pressure; AB: antibodies

Timeline	Clinical Findings and Interventions
Day 1	The patient was put NPO, GI was consulted, and EGD with esophageal manometry was scheduled. *Trypanosoma cruzi* IgG AB was ordered and found to be negative.
Day 2	EGD showed a significant amount of residual food and secretions filling the esophageal and gastric lumen, which was partially cleared. The esophagus was severely dilated and tortuous. The GEJ was located at 45 cm from the incisors and appeared open with no resistance to traverse the gastroscope (OD 12 mm). EGD and HrEM scheduled two days later. Repeat *T. cruzi* IgG AB ordered and found to be negative.
Day 3	Barium esophagogram ordered and showed evidence of achalasia with markedly dilated tortuous esophagus with an abrupt transition point at the level of the GEJ.
Day 4	Similar findings as prior EGD. HrEM with absent contractility in 100% of swallows with no evidence of panesophageal pressurization. Median IRP of 24 mmHg. Impression of Type 1 achalasia. The patient was scheduled for EGD with GEJ dilatation in two days.
Day 6	The patient underwent successful upper GI endoscopy for balloon dilatation.
Day 7	The patient was able to tolerate diet and was successfully discharged.

## Discussion

We present a case of end-stage Type 1 achalasia with megaesophagus in a 51-year-old woman from Nicaragua, a region where Chagas’ disease is endemic [4]. Long-standing, untreated achalasia can lead to progressive neuromuscular degeneration of the esophageal wall, resulting in severe dilation, elongation, and tortuosity - a condition ultimately termed megaesophagus or sigmoid esophagus [1]. Although achalasia most commonly presents with progressive dysphagia, regurgitation, and weight loss, this case is notable for its acute presentation with respiratory distress.

In this patient, severe esophageal dilation produced a substantial mediastinal mass effect likely contributing to her left lower lobe atelectasis via compression of the adjacent lungs or airways. Although an inpatient CT chest was not performed to directly demonstrate this causal relationship, the patient's chest radiograph findings and rapid improvement and resolution of dyspnea after treatment with pneumatic balloon dilation serve as evidence of this assumption. Respiratory manifestations of achalasia, such as nocturnal cough, aspiration, and mild dyspnea, are well-documented; however, acute respiratory compromise from mechanical compression by a massively dilated esophagus is rare. A review of the literature reveals that since 1950, fewer than 70 adult cases of acute respiratory distress secondary to megaesophagus have been reported, primarily affecting older female patients. The mechanical etiology involves a combination of esophageal kinking, high residual upper esophageal sphincter pressure, and a defective belch reflex, which trap air and food inside the lumen [2]. This case highlights the importance of considering advanced esophageal disease when unexplained mediastinal or cardiac silhouette enlargement is seen on chest radiograph.

To navigate the diagnostic complexities of end-stage achalasia and safely exclude critical mimics, a multimodal diagnostic approach is mandatory. Given the patient’s origin from Nicaragua, ruling out secondary megaesophagus from chronic Chagas disease was an important consideration. Current recommendations for the diagnosis of Chagas disease generally require two distinct serologic assays using different antigens or methodologies, as no single assay has adequate sensitivity and specificity across all populations [5]. In this patient, a repeated, non-reactive *T. cruzi* IgG antibody test using a different methodology makes Chagas disease highly unlikely, supporting a diagnosis of idiopathic Type 1 achalasia. Pseudoachalasia was another important consideration as it accounts for up to 4% of patients presenting with achalasia-like symptoms and is most often associated with an occult malignancy involving the gastric cardia or GEJ [6]. In this case, the characteristic manometric findings along with the absence of an obstructing intraluminal lesion on endoscopy made pseudoachalasia less likely. High-resolution esophageal manometry remains the gold standard for confirming achalasia and determining its subtype [1]. The most common finding is an elevated integrated relaxation pressure (IRP) alongside a total absence of peristalsis, as was seen with our patient, and is termed Type 1 or classic achalasia.

Management of end-stage achalasia with megaesophagus can be challenging, and outcome depends on the chronicity and severity of symptoms, esophageal anatomy, and response to treatment. In the acute setting, immediate decompression using a nasogastric tube can effectively alleviate mediastinal mass effect and reverse airway compromise. Temporary treatment options include botulinum toxin injections to paralyze the lower esophageal sphincter and pharmacotherapy with smooth muscle relaxants such as calcium-channel blockers or nitrates. For long-term definitive management, options include laparoscopic Heller myotomy, peroral endoscopic myotomy, pneumatic balloon dilation, or, in refractory cases, esophagectomy. In this patient, pneumatic balloon dilatation successfully restored luminal latency and was followed by rapid improvement in her respiratory symptoms and dysphagia. Her ability to resume a normal oral diet further supported the clinical success of the intervention. This case demonstrates that pneumatic dilation can remain an effective treatment option even in the setting of profound megaesophagus. However, while its initial success is high, it has a high relapse rate; roughly one-third of patients experience recurrent symptoms within four years and require repeat procedures [5]. Therefore, it is often viewed as a step-up approach or an intermediate option rather than a strictly permanent cure.

Long-term follow-up remains important because persistent esophageal retention and stasis can lead to recurrent symptoms, nutritional complications, and an increased risk of esophageal squamous cell carcinoma. The reported interval between the onset of achalasia symptoms and development of esophageal cancer is cited as 10-25 years [4]. Continued clinical follow-up, with objective assessment of esophageal emptying via timed barium esophograms and consideration of periodic upper endoscopy, will therefore be important in this patient to monitor treatment response and identify potential late complications.

## Conclusions

This case demonstrates that end-stage Type 1 achalasia with megaesophagus can present atypically as acute respiratory distress secondary to mediastinal mass effect. When evaluating unexplained mediastinal enlargement or airway compression on chest radiograph, clinicians must maintain a high index of suspicion for esophageal pathology. If achalasia is suspected, secondary causes of achalasia and critical mimics like pseudoachalasia must be ruled out using a multi-modal diagnostic approach. High-resolution esophageal manometry remains the gold standard to definitively anchor the diagnosis by confirming an elevated IRP alongside a total absence of contractility. This case reinforces that pneumatic balloon dilation can remain a highly effective intermediate option even in profound megaesophagus, successfully restoring luminal patency and resolving secondary compressive respiratory symptoms. Ultimately, long-term follow-up is essential to monitor treatment response and identify potential late complications.
